# How stressful life events are associated with depression: the mediating pathway of security in a clinical adolescent sample

**DOI:** 10.3389/fpsyt.2026.1802678

**Published:** 2026-04-14

**Authors:** Liqi Gu, Xin Tian, Xinrong Ma, Ling Dang, Yuelan Zhang, Mengyuan Zhang, Furong Gou

**Affiliations:** 1 School of Nursing, Ningxia Medical University, Ningxia, China; 2 Shanxi Bethune Hospital, Shanxi Academy of Medical Sciences, Third Hospital of Shanxi Medical University, Tongji Shanxi Hospital, Taiyuan, China; 3 Department of Psychiatry and Clinical Psychology, Ningxia Ning-An Hospital, Ningxia Mental Health Center, Ningxia, China

**Keywords:** adolescent, depression, dual-pathway model, mediation, security, stressful life events

## Abstract

**Background:**

Stressful life events are well-established risk factors for adolescent depression; however, the psychological mechanisms underlying this association remain insufficiently understood, particularly regarding which types of stress and which dimensions of security are most closely linked to depression. This study aimed to investigate whether security and its two sub-dimensions statistically mediated the association between stressful life events and depression among clinically diagnosed adolescents, while also examining the relative strength of indirect associations across specific stress types.

**Methods:**

A cross-sectional study was conducted with 284 adolescents (70.1% female; mean age = 15.82 ± 1.86 years) diagnosed with major depressive disorder according to the DSM-5 criteria at a tertiary psychiatric hospital in Western China. Participants completed the Adolescent Self-Rating Life Events Checklist (ASLEC), Self-Rating Depression Scale (SDS), and Security Questionnaire (SQ) questionnaires. Simple mediation, parallel mediation, and dimension-specific analyses were performed using the PROCESS macro (Model 4) with 5,000 bootstrap resamples, controlling for gender and parental marital status.

**Results:**

stressful life events were significantly positively correlated with depression (r = 0.491, p < 0.001) and negatively correlated with security (r = −0.464, p < 0.001). Simple mediation analysis revealed that security demonstrated a significant indirect association through security (indirect effect = 0.176, 95% CI [0.126, 0.232]), accounting for 53.8% of the total association. Parallel mediation analysis further indicated a dual-pathway model: both Interpersonal Security (indirect effect = 0.083, 95% CI [0.037, 0.133]) and Certainty in Control (indirect effect = 0.093, 95% CI [0.043, 0.152]) functioned as significant statistical mediators of comparable magnitude, with no significant difference between them (Contrast = −0.010, 95% CI [−0.065, 0.042]). Furthermore, dimension-specific analyses revealed that Interpersonal Stress (standardized indirect effect = 0.266) and Academic Stress (standardized indirect effect = 0.231) showed the strongest indirect associations with depression through the security pathway. Exploratory subgroup analyses revealed a gender-crossed pattern: for male adolescents (n = 85), the indirect association was significant only through Interpersonal Security (effect = 0.116, 95% CI [0.048, 0.199]); for female adolescents (n = 199), it was significant only through Certainty in Control (effect = 0.136, 95% CI [0.067, 0.212]).

**Conclusion:**

Security functions as a significant statistical mediator in the association between stressful life events and adolescent depression. The findings are consistent with a “dual-pathway” model wherein stress is concurrently associated with lower levels of both relational security (Interpersonal Security) and personal agency (Certainty in Control). Exploratory analyses suggest that the relative importance of these two pathways may differ by gender. If confirmed by future longitudinal research, clinical interventions may benefit from an integrated approach that addresses both dimensions, with particular attention to interpersonal conflicts and academic pressure as the stressors most strongly associated with depression through security pathways.

## 1 Introduction

Adolescence is an important time when young people go through significant changes in their bodies, minds, and emotions, during which the risk of psychological disorders, particularly depression, markedly increases (1). A comprehensive review revealed that the worldwide occurrence of mental health issues in children and teenagers is 13.4%, and the prevalence of depressive disorders is 2.6% (2). Significantly, the prevalence of depressive disorders among adolescents in China has risen to 24.3%, which is considerably greater than that found in other nations (3), and the prevalence is greater in females than in males (4). Symptoms of depression negatively impact adolescents’ academic performance and social interactions, potentially leading to both short-term and long-term behavioral issues (5, 6), and may even be associated with the occurrence of non-suicidal self-injury and suicidal behaviors (7, 8).

According to stress sensitization theory, exposure to stressful life events during critical developmental periods may heightened individual’s vulnerability to subsequent stressors, thereby being associated with an increased risk of depression (9). Life events that are associated with stress are identified as major risk factors for depression in adolescents, as they are associated with intensified feelings of sadness and reduce overall well-being (10). Stressful life events, including academic pressure, interpersonal conflicts, and family changes, are consistently observed as risk factors for depression in adolescents (11–13). Specifically, recent stressful life events, such as school stress and problems with friends are associated with depressive symptoms, with a cumulative effect (14). The relationship between stressful life events and depression is characterized by complexity. Specifically, individuals experiencing elevated levels of stressful life events tend to demonstrate an association with more pronounced depressive symptoms (15). Moreover, an increase in depressive symptoms has been suggested to be associated with a higher incidence of stressful life events that are dependent on interpersonal issues (16). However, Liu et al. suggest that experiencing stress early in life may enhance an individual’s capacity to handle stress encountered in the future (17). This finding implies that the relationship between stress on human growth should not be considered purely harmful; instead, it could be shaped by a range of psychological or behavioral influences. In this context, it is particularly noteworthy that other mediating mechanisms in the stress-depression relationship require further investigation, especially in adolescents with clinical depression.

Grounded in Maslow’s hierarchy of needs, security pertains to an individual’s subjective perception of security and stability concerning potential threats to their physical or psychological well-being within interpersonal and social contexts (18). Individuals with higher security tend to exhibit greater self-confidence and emotional stability, whereas those with lower security are more susceptible to anxiety, fear, and depression (19). The theory of emotional security posits that a child’s sense of security is a key factor in emotional and behavioral regulation (20). The establishment of security relies on the fulfillment of individual-level needs (including social support and a perception of physical security) and organizational environments (such as non-punitive management) (21). Research indicates that stressful life events are associated with the erosion of an individual’s attachment, thereby potentially weakening their sense of security (22), and a lack of security can be linked to the undermining of security (23). Furthermore, traumatic experiences are often associated with a diminution of an individual’s long-term security (24). Additionally, another cross-sectional study found that the more stressful life events one experiences, the more severe the symptoms of depression become, and there is a correlation between decreased security and exacerbated depressive symptoms (25). While security is often treated as a unitary construct, it comprises two distinct dimensions: interpersonal security (feeling safe in relationships) and sense of certainty and control (feeling in control of one’s life). It remains unclear whether stressful life events are related to depression predominantly through associations with damage to interpersonal connections or through associations with the erosion of the individual’s Certainty in Control over their environment. Differentiating between these pathways is crucial for identifying precise targets for clinical intervention.

Therefore, we aimed to examine whether security is statistically consistent with an indirect association between stressful life events and depression in adolescents with clinically diagnosed depression. While previous research has explored security as a unitary construct in community samples, our study extends this understanding by distinguishing its two core dimensions—Interpersonal Security and Certainty in Control—and testing their associations in a clinical major depressive disorder sample. This distinction is important because these dimensions may reflect distinct patterns of vulnerability. Furthermore, given the multidimensional nature of adolescent stress, we examine whether different types of stressful life events (e.g., academic vs. interpersonal) are differentially associated with these security dimensions, offering a more nuanced view than prior work. By focusing on these associations in a clinically diagnosed population, our study aims to clarify how stressful experiences are associated with levels of specific security dimensions, which are concurrently associated with depressive symptomatology. thereby informing more targeted intervention efforts. Accordingly, based on our cross-sectional model, we propose the following hypotheses: Hypothesis 1 (H1): Security will demonstrate a significant indirect association between stressful life events and depression. Hypothesis 2 (H2): Both Interpersonal Security and Certainty in Control are observed to serve as significant parallel mediators in this association. Hypothesis 3 (H3): The two security dimensions may exhibit differential indirect associations. Hypothesis 4 (H4): Specific dimensions of stressful life events, particularly interpersonal and academic stress, will exhibit the strongest indirect associations with depression through their link to levels of security.

## 2 Materials and methods

### 2.1 Participants

This study adopted a cross-sectional design, involving 284 adolescents who were admitted to the Pediatrics and Adolescent Department at Ning’an Hospital in the Ningxia Hui Autonomous Region of China, during the period from March 2024 to February 2025. All participants were screened for depression by at least two psychiatrists using the diagnostic criteria outlined in the DSM-5. The criteria for inclusion were as follows (1) Individuals aged 12–18 years. (2) Those who fulfilled the diagnostic requirements for major depression, as specified in the DSM-5. 3. Ability to understand and complete the questionnaire. The criteria for exclusion were as follows: (1) presence of severe comorbid mental or neurological disorders, (2) Organic diseases, (3) Cognitive impairments that could affect the assessment. Prior to this, the surveyors had completed a standardized training program that focused on data collection. This study was approved by the hospital ethics committee (Approval No: KYSC202300033; Ethics No 2023-WS-022). Fritz and MacKinnon suggest that detecting moderate or small mediation association generally necessitates a sample size of 200 or more to ensure adequate statistical power (26). Consequently, the sample size of 284 used in this study is sufficient and aligns with the recommended standards for mediation analysis involving multiple variables.

### 2.2 Measures

#### 2.2.1 General information

Based on the research objectives and literature review, a general information survey was designed to investigate factors such as gender, age, education level, only child status, and parental marital status among adolescents with depression.

#### 2.2.2 The adolescent life events scale

The adolescent life events scale, comprising 27 items, was used to evaluate the stressful experiences encountered by teenagers during the previous six months. To ensure cross-cultural rigor, we utilized the version originally developed and validated specifically for Chinese adolescents by Liu et al. (27), which inherently accounts for the Chinese cultural context. Its robust psychometric properties in Chinese populations have been widely confirmed in recent studies (28). These events encompass academic pressures, interpersonal conflicts, and family changes. A five-point scoring system was used, with a higher score indicating increased pressure. In this study, Cronbach’s α was 0.89.

#### 2.2.3 The security questionnaire

The security questionnaire was designed to assess individuals’ sense of security. This scale was natively compiled by Chinese scholars to specifically assess the psychological security of the Chinese population, thereby inherently aligning with the Chinese cultural background (29). Recent studies have confirmed its good reliability and validity in Chinese adolescents (30). Employing a five-point Likert scale, higher scores indicate a stronger sense of security. In this study, the overall Cronbach’s α for the scale was 0.91; specifically, the Cronbach’s α for the ‘Interpersonal security’ dimension was 0.88, and for the ‘Sense of Control’ dimension, it was 0.86. This questionnaire consists of 16 self-assessment items aimed at measuring these two dimensions. In the ‘Interpersonal security’ dimension, relevant items include ‘I never dare to express my opinions actively’; in the ‘Sense of Control’ dimension, relevant items include ‘I feel that my life is full of uncertainty’.

#### 2.2.4 The self-rating depression scale

The self-rating depression scale developed by Zung in 1965 functions as a self-evaluation instrument aimed at assessing the intensity of depressive conditions and their variations during treatment (31). In this study, we utilized the widely adapted Chinese version of the SDS, which has undergone formal validation and demonstrated sound psychometric properties and cultural adaptability in Chinese populations (32). The instrument consists of 20 items, each rated on a scale from one to four. Within this collection, 10 items were positively phrased (items 2, 5, 6, 11, 12, 14, 16, 17, 18, and 20) and scored in reverse. The Self-Rating Depression Severity Index was determined by dividing the total score of all items by 80, the highest possible score, which produced an index ranging from 0.25 to 1.0. A higher score on this index indicates greater depression severity. In the context of this study, Cronbach’s α was 0.92, indicating strong reliability.

### 2.3 Procedures

After obtaining ethical approval, the researchers contacted clinicians at a psychiatric specialty hospital to identify eligible adolescents diagnosed with depression. Following the acquisition of informed consent from both the adolescents and their guardians, trained researchers guided the participants to complete the questionnaires in a quiet environment. The entire process lasted approximately 30–40 min. To protect the participants’ privacy, all data were made anonymized. The researchers collected 300 questionnaires using a continuous sampling method. Among them, 16 questionnaires were discarded due to invalid criteria, accounting for 5.3% of the initial sample. These invalid questionnaires included those with more than 10% missing responses on any scale (e.g., the Depression Scale, the Security Questionnaire), responses that exhibited clear contradictions (such as selecting the same answer for all questions or demonstrating illogical response patterns), and those missing crucial demographic information required for analysis. After excluding invalid questionnaires, 284 valid questionnaires were included in the analysis, resulting in a valid response rate of 94.67%. In the sample, males accounted for 29.9%, while females accounted for 70.1%. The age range of the participants was between 12 and 18 years (M ± SD=15.82 ± 1.86).

### 2.4 Data analysis

We conducted our statistical analysis using SPSS version 27.0 and the PROCESS plugin version 4.1, following a four-step approach. First, we checked for common method bias with Harman’s single-factor test, which suggests that the variance of the first common factor should not exceed 40% (33). In the next step, we summarized participant differences using means and standard deviations. The third phase involved correlation analysis to explore the relationships between variables. Subsequently, we performed mediation analysis using the bootstrap method with 5000 resamples (Model 4) through the PROCESS plugin, employing a parallel multiple mediation model to simultaneously assess specific indirect association. Pairwise contrasts were used to compare the size of the indirect effects across different dimensions. A 95% bootstrap confidence interval that excluded zero was considered statistically significant, with a p-value of <0.05 deemed significant for further analysis. Throughout the analyses, demographic factors were included as covariates. We controlled for gender and parental marital status because (1) research shows significant sex differences in adolescent depression, and (2) parental marital status is associated with adolescents’ stress exposure and psychological adjustment; our preliminary analyses also indicated significant differences in depression and security across marital-status groups. In addition, we examined other demographic variables available in this data-set (e.g., age, only-child status, and education), but they were not significant and did not materially change the focal estimates; therefore, to maintain parsimony, they were not retained in the final models.

## 3 Results

### 3.1 Common method bias analysis

A common method bias evaluation was performed using the Adolescent Life Events Scale, Self-Rating Depression Scale, security Questionnaire. The findings revealed that 17 factors with eigenvalues exceeding one were identified without any rotation, with the first factor explaining 26.55% of the variance (less than 40%), which implies a minimal impact of common method bias on the outcomes of this research.

### 3.2 Demographic characteristics

Participant characteristics are summarized in **Table 1**. The sample included 284 adolescents, of whom 199 were female (70.1%) and 85 were male (29.9%). The mean age was 15.82 years (SD = 1.86). Sex differences were observed for both depression and security: female participants reported higher depression scores than males (M = 63.39 vs. 54.01, p < 0.001) and lower security (M = 41.25 vs. 46.19, p < 0.01). Adolescents from divorced households reported higher levels of stressful life events and depression than those from intact families (p < 0.05), and they also reported lower security.

**Table 1:** Demographic characteristics and group differences in study variables (N = 284).

Variables	N(%)	Stressful life events (M±SD)	t or F	Depression (M±SD)	t or F	Security (M±SD)	t or F
Gender			-0.983		-4.912***		2.952**
Male	85 (29.9)	56.00 (22.34)		54.01 (13.82)		46.19 (11.71)	
Female	199 (70.1)	58.76 (21.39)		63.39 (15.10)		41.25 (13.39)	
Age			1.649		2.724		2.424
12-14	62 (21.8)	50.96 (18.89)		51.36 (14.32)		51.36 (14.32)	
15-16	138 (48.6)	58.15 (20.83)		39.83 (14.27)		39.83 (14.27)	
17-18	84 (29.6)	54.62 (18.60)		42.79 (11.92)		42.79 (11.92)	
Only Child Status			1.347		1.559		-1.971
Only Child	183 (64.4)	59.22 (22.09)		61.58 (16.17)		41.60 (12.88)	
With Sibings	101 (35.6)	55.60 (20.81)		58.77 (13.53)		44.78 (13.26)	
Education			1.765		2.661		2.363
Primary School	14 (4.9)	49.15 (19.03)		51.14 (10.26)		51.57 (16.85)	
Middle School	115 (40.5)	59.93 (23.35)		62.30 (15.81)		42.74 (13.48)	
High School	125 (44.0)	58.39 (20.42)		60.74 (15.00)		41.91 (11.98)	
College	30 (10.6)	52.45 (20.25)		57.70 (15.30)		41.97 (13.19)	
Parental Marital Status			-2.784**		-2.743**		1.957
Married	250 (88.0)	56.62 (21.31)		59.67 (15.04)		43.28 (13.02)	
Divorced	34 (12.0)	67.53 (22.25)		67.26 (15.91)		38.63 (13.05)	

M, Mean; SD, Standard Deviation. Differences tested via t-test or F-test. **P<0.01, ***p <0 .001.

### 3.3 Correlation analysis

**Table 2** presents correlations among stressful life events, security, and depression (N = 284). Stressful life events were positively correlated with depression (r = 0.491, p < 0.01). Security was negatively correlated with depression (r = −0.691, p < 0.01) and with stressful life events (r = −0.464, p < 0.01). These correlations indicate that greater stressful life events co-occurred with higher depression and lower security, and that lower security co-occurred with higher depression.

**Table 2:** Correlations among the measured variables(N=284).

Variables	Mean	SD	1	2	3
Stressful Life Events	57.93	21.67	1		
Depression	60.58	15.32	.491**	1	
Security	42.72	13.09	-.464**	-.691**	1

**P<0.01, ***P<0.00.1.

### 3.4 Regression result for the mediation model

Regression results are summarized in **Table 3**. In Model 1, the overall model was significant (F = 29.11, p < 0.001) and explained 23.8% of the variance (R² = 0.238). Stressful life events were significantly negatively associated with security (b = −0.271, SE = 0.032, t = −8.47, p < 0.001). In Model 2, including both stressful life events and security yielded a significant model (F = 83.94, p < 0.001) explaining 54.6% of the variance in depression (R² = 0.546). Security was negatively associated with depression (b = −0.651, SE = 0.054, t = −12.03, p < 0.001). Stressful life events remained positively associated with depression after including security (b = 0.151, SE = 0.032, t = 4.66, p < 0.001). Gender was also significant, with females reporting higher depression (b = 5.692, SE = 1.368, t = 4.16, p < 0.001).

**Table 3:** Regression analysis results for the mediation model (N = 284).

Outcome variable	Predictive variable	R2	F	b	β	SE	t	95% CI
Model 1								
Security	Stressful Life Events	0.238	29.107	-0.271	-0.449	0.032	-8.473**	[-0.334, -0.208]
	Gender			4.160	0.146	1.491	2.790**	[1.225, 7.095]
	Parental Status			-1.541	-0.038	2.129	-0.724	[-5.731, 2.649]
Model 2								
Depression	Stressful Life Events	0.546	83.939	0.151	0.214	0.032	4.662	[0.087, 0.215]
	Security							[-0.757, -0.544]
	Gender							[-8.385, -2.999]
	Parental Status							[-1.105, 6.485]

**P<0.01. CI, Confidence Interval. b, Unstandardized Coefficient. β, Standardized Coefficient.

### 3.5 Mediation analysis of stressful life events on depression via security

**Figure 1:**
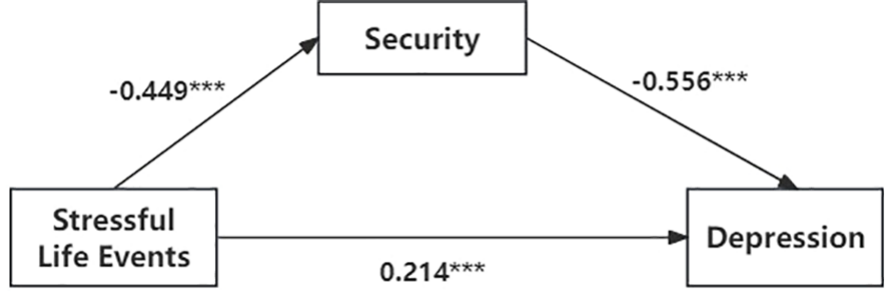
A cross-sectional mediation model of security between stressful life events and depression.

The simple mediation analysis (PROCESS Model 4) supported H1. As shown in **Table 4** (see also **Figure 1**), stressful life events showed a significant total association with depression (b = 0.328, β = 0.463, p < 0.001). After controlling for gender and parental marital status and including security as the mediator, the direct association between stressful life events and depression remained significant (b = 0.151, β = 0.214, p < 0.001). The indirect association via security was also significant (b = 0.176, β = 0.249), accounting for 53.8% of the total association. These results indicate that security statistically accounted for a substantial portion of the stressful life events–depression association in this cross-sectional sample.

**Table 4:** Mediation analysis of stressful life events on depression through security (N = 284).

	b	SE	95% CI	Mediated%	β	95% CI
Total effect	0.328	0.036	[0.257, 0.398]	–	0.463	–
Direct effect	0.151	0.032	[0.087, 0.215]	46.2%	0.214	–
Indirect effect	0.176	0.027	[0.126, 0.232]	53.8%	0.249	[0.182, 0.322]

CI, Confidence Interval; b, Unstandardized Coefficient; β, Standardized Coefficient.

### 3.6 Parallel mediation analysis: two security dimensions

**Figure 2:**
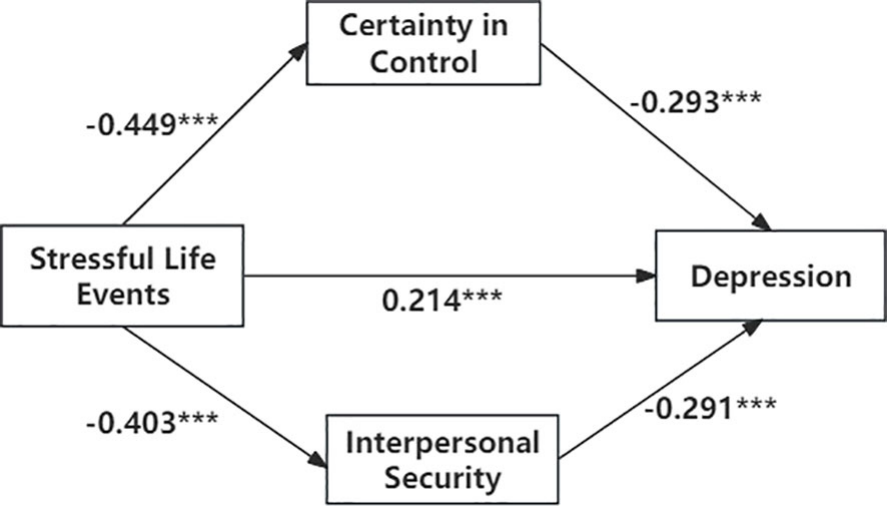
A cross-sectional mediation model of interpersonal security and certainty in control in the relationship between stressful life events and depression. *** P < 0.001.

Parallel mediation results are shown in **Table 5** (see also **Figure 2**). Both Interpersonal Security and Certainty in Control showed significant indirect associations in the link between stressful life events and depression. The total indirect association through the two dimensions was significant (b = 0.176, β = 0.249). Specifically, the indirect association through Interpersonal Security was significant (b = 0.083, β = 0.117), and the indirect association through Certainty in Control was also significant (b = 0.093, β = 0.132). These findings support H2. For H3, the contrast between the two specific indirect associations was not significant (contrast = −0.010), indicating insufficient evidence that one indirect association was stronger than the other in this cross-sectional data-set.

**Table 5:** Parallel mediation analysis: effects of total stressful life events on depression via two security dimensions (N = 284).

	b	SE	95% CI	β	95% CI
Total Effect	0.328	0.036	[0.25, 0.398]	0.463	–
Direct Effect	0.152	0.033	[0.088, 0.216]	0.214	–
Indirect Effect	0.176	0.028	[0.125, 0.234]	0.249	[0.181, 0.323]
Certainty in control	0.083	0.025	[0.037, 0.133]	0.117	[0.052, 0.185]
Interpersonal Security	0.093	0.028	[0.043, 0.152]	0.132	[0.061, 0.214]
Contrast(Certainty in control- Interpersonal Security)	-0.010	0.028	[-0.065, 0.042]	–	–

CI, Confidence Interval; b, Unstandardized Coefficient; β, Standardized Coefficient.

### 3.7 Supplementary analyses: gender differences in the mediation model

Given the over-representation of female participants (70.1%) and known gender differences in stress responses and depression, we examined whether the indirect associations differed by gender. The mediation model (PROCESS Model 4) was run separately for male (n = 85) and female (n = 199) adolescents, controlling for parental-related variables. Following Hayes, we use the terms “direct effect” and “indirect effect” as standard statistical terms; they do not imply causality in this cross-sectional study. Results are shown in **Tables 6**, **7**. For male adolescents, stressful life events were negatively associated with both interpersonal security (b = -0.097, β = -0.366) and certainty in control (b = -0.110, β = -0.371). In the depression model, interpersonal security was strongly associated with depression (b = -1.199, β = -0.513), but certainty in control was not (b = -0.219, β = -0.105). The indirect association through interpersonal security was significant (β = 0.187, 95% CI [0.048, 0.199]), while the one through certainty in control was not (β = 0.081, 95% CI [-0.031, 0.113]). For female adolescents, stressful life events were also significantly associated with both interpersonal security (b = -0.135, β = -0.425) and certainty in control (b = -0.163, β = -0.482). In the depression model, certainty in control showed a stronger association with depression (b = -0.837, β = -0.400), while the association between interpersonal security and depression was weaker (b = -0.426, β = -0.191). The indirect association through certainty in control was significant (β = 0.193, 95% CI [0.067, 0.212]), while the one through interpersonal security was not (β = 0.081, 95% CI [-0.006, 0.126]).

**Table 6:** Parallel mediation model results by gender: direct path coefficients.

		Male (n=85)					Female (n=199)				
Outcome variable	Predictive variable	b	SE	t	β	95% CI	b	SE	t	β	95% CI
Model 1											
Interpersonal Security	Stressful Life Events	-0.097	0.028	-3.347**	-0.366	[-0.153, -0.041]	-0.135	0.021	-6.530***	-0.425	[-0.176, -0.094]
Model 2											
Certainty in Control	Stressful Life Events	-0.110	0.029	-3.792***	-0.371	[-0.168, -0.052]	-0.163	0.021	-7.631***	-0.482	[-0.205, -0.121]
Model 3											
Depression	Interpersonal Security	-1.199	0.288	-4.165***	-0.513	[-1.772, -0.626]	-0.426	0.199	-2.135*	-0.191	[-0.819, -0.033]
	Certainty in Control	-0.219	0.279	-0.785	-0.105	[-0.773, 0.336]	-0.837	0.193	-4.331***	-0.400	[-1.219, -0.456]
	Stressful Life Events	0.137	0.054	2.561	0.221	[0.031, 0.243]	0.157	0.041	3.866***	0.223	[0.077, 0.237]

*P<0.05,**P<0.01, ***P<0.001. CI, Confidence Interval; b, Unstandardized Coefficient; β, Standardized Coefficient.

**Table 7:** Effects and pairwise contrasts by gender.

	Male (n=85)				Female (n=199)			
	B	SE	β	95% CI	b	SE	β	95% CI
Total Effect	0.277	0.062	0.448	[0.154, 0.400]	0.351	0.044	0.497	[0.265, 0.437]
Indirect Effect	0.140	0.044	0.226	[0.122, 0.350]	0.194	0.033	0.274	[0.191, 0.365]
Via Interpersonal Security	0.116	0.039	0.187	[0.048, 0.199]	0.057	0.033	0.081	[-0.006, 0.126]
Via Certainty in Control	0.024	0.037	0.039	[-0.031, 0.113]	0.136	0.037	0.193	[0.067, 0.212]
Pairwise Contrast	0.092	0.061	0.149	[-0.059, 0.330]	-0.079	0.062	-0.111	[-0.290, 0.055]

CI, Confidence Interval; b, Unstandardized Coefficient; β, Standardized Coefficient.

### 3.8 Specificity analysis: stress dimensions and depression via security

To examine H4 and identify stress domains most strongly linked to depression via security, we estimated mediation models for six stressful life event dimensions (**Table 8**). All six stress dimensions showed significant indirect associations with depression through overall security, although the magnitudes differed. The strongest indirect association was observed for interpersonal stress (β = 0.266), followed by academic stress (β = 0.231). These were larger than those for punitive stress (β = 0.215), health adaptation stress (β = 0.198), and loss-related stress (β = 0.167). These results support H4, indicating that interpersonal and academic stress showed the strongest indirect associations with depression through their associations with lower security.

**Table 8:** Specificity analysis: indirect effects of different stress dimensions on depression via security (N = 284).

Stress dimension	b	SE	95% CI	Mediated%	β	95% CI
Total effect	0.328	0.036	[0.257, 0.398]	–	0.463	[0.257, 0.398]
Direct effect	0.151	0.032	[0.087, 0.215]	–	0.214	[0.087, 0.215]
Indirect Effect	0.176	0.028	[0.125, 0.234]	53.6%	0.249	[0.182, 0.322]
Interpersonal	0.727	0.105	[0.532, 0.947]	55.1%	0.266	[0.198, 0.341]
Academic	0.741	0.122	[0.516, 0.994]	57.1%	0.231	[0.160, 0.303]
Punishment	0.574	0.101	[0.393, 0.786]	55.8%	0.215	[0.146, 0.288]
Health adaptation	0.965	0.196	[0.629, 1.373]	61.3%	0.198	[0.134, 0.270]
Loss	0.919	0.192	[0.526, 1.302]	55.7%	0.167	[0.170, 0.309]
Other	1.010	0.168	[0.705, 1.365]	55.7%	0.236	[0.096, 0.237]

CI, Confidence Interval; b, Unstandardized Coefficient; β, Standardized Coefficient.

## 4 Discussion

This study examined cross-sectional associations among stressful life events, security, and depression in clinically diagnosed adolescents and evaluated whether security statistically accounted for (i.e., was indirectly associated with) the stressful life events–depression association. Three main findings emerged: (1) security accounted for a substantial portion (53.8%) of the total association between stressful life events and depression; (2) Interpersonal Security and Certainty in Control both showed significant parallel indirect associations, with no evidence that one was stronger than the other; and (3) interpersonal and academic stress exhibited the strongest indirect associations with depression via security. These findings refine how stress, security, and depression co-occur in this clinical sample, while not establishing temporal or causal direction.

### 4.1 Indirect association via overall security

A central finding was that the association between stressful life events and depression was largely accounted for by security, with over half of the total association occurring through this indirect pathway. This pattern is consistent with prior work in community adolescent samples. For example, Niu et al. (34). and Cao et al. (35). reported that security statistically mediated associations involving cyberbullying, adjustment, and depression. The present study extends these observations to a clinically diagnosed major depressive disorder sample. The relatively large indirect proportion observed here may reflect that clinically depressed adolescents tend to report lower security, which could strengthen the co-occurrence between stress exposure, reduced security, and depressive symptoms. From the perspective of conservation of resources theory, threats to valued psychological resources are associated with greater distress (36). Security may be conceptualized as such a resource, reflecting perceived predictability, coping capacity, and interpersonal trust (37). Accordingly, adolescents reporting more stressful events may also report lower security, which co-occurs with higher depressive symptoms (38). Related evidence likewise indicates that greater psychological resources are associated with lower depression risk (39).

### 4.2 Parallel indirect associations of certainty in control and interpersonal security

Decomposing security into Certainty in Control and Interpersonal Security, we found that both dimensions showed significant indirect associations in parallel, and the contrast between their specific indirect effects was not significant. This non-significant contrast does not establish equivalence; rather, it indicates insufficient evidence to conclude that one indirect association is stronger than the other. The pattern suggests that stressful life events co-occur with both reduced perceived control and reduced interpersonal security, and that both are linked to depressive symptoms (40). Although we initially expected Interpersonal Security to show a stronger indirect association (41), adolescence is characterized by salient needs for belonging and peer acceptance (42), while clinical depression is often accompanied by both helplessness and loneliness (43). Prior literature links repeated uncontrollable experiences to learned helplessness processes relevant to depression (44), and relational trauma to disengagement or “giving up” (45). Conceptually, Interpersonal Security aligns with attachment frameworks emphasizing relational safety and acceptance (46), whereas certainty in control aligns with perceived control/self-efficacy and helplessness–hopelessness models of depression (47). These perspectives also allow for interplay: relational insecurity may undermine perceived agency, and low perceived control may promote interpersonal withdrawal, with both patterns co-occurring in adolescent depression.

### 4.3 Gender-specific patterns in indirect associations

The exploratory subgroup analyses revealed a crossover pattern. For male adolescents, stressful life events were linked to depression only through interpersonal security. For female adolescents, the link was only through certainty in control. Because our data is cross-sectional, we cannot prove cause and effect, but these results suggest that boys and girls process security differently. For males, interpersonal security was the main link. People often assume boys care more about independence than relationships. However, society often tells boys not to show emotions or ask for help (48). Because of this, when boys face stress, they may lack emotional support, making them highly vulnerable to depression if their relationships feel unsafe (49). This matches recent studies showing that poor relationship security is closely tied to emotional problems in boys (50). For females, certainty in control was the main link. This fits with psychological models showing that feeling out of control leads to depression (51). When stressed and feeling out of control, girls are more likely than boys to constantly overthink their problems—a habit called rumination (52, 53). This constant negative thinking is a major risk factor for depression. Furthermore, losing a sense of control strongly hurts adolescents’ overall well-being (54). Therefore, a lack of control may trigger depression more easily in girls.

### 4.4 Stress-dimension specificity

Across six stress domains, interpersonal stress and academic stress exhibited the strongest indirect associations with depression via security. This is consistent with the view that interpersonal difficulties are a core correlate of adolescent depression (42) and with evidence highlighting social connection as central to mental health (55) Academic stress also showed a comparatively large indirect association, which may be particularly relevant in the Chinese cultural context where achievement is closely tied to expectations, self-worth, and social evaluation (56, 57). Prior work suggests academic pressure is a prominent correlate of adolescent mental health concerns in China (58). One interpretation is that academic stress co-occurs with both lower perceived control and heightened concerns about relational evaluation, both of which are linked to depressive symptoms.

### 4.5 Clinical implications

In terms of practical implications, although our data are cross-sectional and cannot support causal inference, the findings still point to actionable targets for adolescent depression care. In line with a comprehensive, multi-setting approach efforts may include: strengthening family and school and community support, providing brief, structured coping stress management support for common stressors (especially interpersonal and academic stress) and offering evidence-based psychological interventions (e.g., cognitive behavioral therapy) to improve emotion regulation and problem solving (59). Together, these measures may help reduce depressive symptom burden by enhancing adolescents’ perceived security and support resources.

## 5 Limitations

This study has several limitations. First, the cross-sectional, self-report design precludes conclusions about temporal order or causality; the tested mediation model reflects patterns of association rather than causal processes. Reverse or reciprocal pathways are plausible (e.g., depressive cognitive biases may influence recall of stressful events and perceptions of relational security and control). Future research should use multi-wave longitudinal designs to test whether stressful events temporally precede changes in security, which in turn precede changes in depression, and should incorporate multi-informant/behavioral indicators to reduce common method variance and social desirability bias. Second, the sample was gender-imbalanced (70.1% female), and the male sub-sample (n = 85) provided limited power, which may partly explain the non-significant indirect association via certainty in control among males. Because these subgroup findings were exploratory, they should be treated as hypothesis-generating; future studies should recruit gender-balanced samples and use multi-group SEM to formally test gender differences. Third, some clinically relevant covariates (e.g., depression severity, medication status) were not adequately captured due to privacy concerns and participant burden, leaving potential unmeasured confounding. Finally, although participants were recruited from Western China, China’s nationwide emphasis on academic achievement and the National College Entrance Examination system may heighten the salience of academic stressors; therefore, generalizability may be limited in contexts where academic competition is less central. Cross-cultural replications and measurement in-variance testing are needed to evaluate robustness across cultures.

## 6 Conclusions

This study confirms that security significantly mediates the relationship between stressful life events and adolescent depression, accounting for 53.8% of the total association. Our findings support a “dual-pathway” model, where stress impacts depression through both diminished interpersonal security and certainty in control. Interpersonal and academic stress were identified as key drivers within this model. Gender subgroup analyses revealed distinct pathways: Interpersonal security was the primary mediator for males, while certainty in control was dominant for females. These findings suggest that clinical interventions could be gender-tailored, focusing on relational security for boys (e.g., family therapy) and personal agency/control for girls (e.g., cognitive behavioral therapy), while prioritizing interpersonal conflicts and academic pressures for both.

## Data Availability

The raw data supporting the conclusions of this article will be made available by the authors, without undue reservation.
